# Phage-Mediated Immune Evasion and Transmission of Livestock-Associated Methicillin-Resistant *Staphylococcus aureus* in Humans

**DOI:** 10.3201/eid2611.201442

**Published:** 2020-11

**Authors:** Raphael N. Sieber, Tinna R. Urth, Andreas Petersen, Camilla H. Møller, Lance B. Price, Robert L. Skov, Anders R. Larsen, Marc Stegger, Jesper Larsen

**Affiliations:** Statens Serum Institut, Copenhagen, Denmark (R.N. Sieber, T.R. Urth, A. Petersen, C.H. Møller, R.L. Skov, A.R. Larsen, M. Stegger, J. Larsen);; Translational Genomics Research Institute, Flagstaff, Arizona, USA (L.B. Price, M. Stegger);; George Washington University, Washington, DC, USA (L.B. Price)

**Keywords:** methicillin-resistant *Staphylococcus aureus*, MRSA, LA-MRSA, humans, livestock, zoonoses, bacteriophages, immune evasion, disease transmission, bacteria, antimicrobial resistance, Denmark, MRSA and other staphylococci, SCIN, CHIPS, SAK, SEA, SEP

## Abstract

Livestock-associated methicillin-resistant *Staphylococcus aureus* (LA-MRSA) can acquire phage-encoded immune modulators, such as the immune evasion cluster (IEC), which protects bacteria from components of the human innate immune system, and the enzyme TarP, which protects against antibody-mediated immune recognition. We used whole-genome sequencing and epidemiologic investigations to study the effects of IEC- and *tarP*-harboring phages on household transmission of LA-MRSA in North Denmark Region during 2004–2011. We reviewed information about all patients throughout Denmark who experienced LA-MRSA infection during 2007–2018 to determine whether IEC is associated with increased spread into the general population. Horizontal acquisition of IEC in the human host was associated with increased household transmission of LA-MRSA and spillover into the community and healthcare settings, whereas we found no evidence to suggest that IEC-positive LA-MRSA isolates have become self-sustainable in the general population. By contrast, TarP did not seem to influence household transmission of LA-MRSA.

## Abstract 2

These bacteria are capable of adapting to humans, leading to increased spread into the community and healthcare settings.

## Medscape CME ACTIVITY

In support of improving patient care, this activity has been planned and implemented by Medscape, LLC and Emerging Infectious Diseases. Medscape, LLC is jointly accredited by the Accreditation Council for Continuing Medical Education (ACCME), the Accreditation Council for Pharmacy Education (ACPE), and the American Nurses Credentialing Center (ANCC), to provide continuing education for the healthcare team.

Medscape, LLC designates this Journal-based CME activity for a maximum of 1.00 ***AMA PRA Category 1 Credit(s)*™**. Physicians should claim only the credit commensurate with the extent of their participation in the activity.

Successful completion of this CME activity, which includes participation in the evaluation component, enables the participant to earn up to 1.0 MOC points in the American Board of Internal Medicine's (ABIM) Maintenance of Certification (MOC) program. Participants will earn MOC points equivalent to the amount of CME credits claimed for the activity. It is the CME activity provider's responsibility to submit participant completion information to ACCME for the purpose of granting ABIM MOC credit.

All other clinicians completing this activity will be issued a certificate of participation. To participate in this journal CME activity: (1) review the learning objectives and author disclosures; (2) study the education content; (3) take the post-test with a 75% minimum passing score and complete the evaluation at http://www.medscape.org/journal/eid; and (4) view/print certificate. 

### Release date: October 16, 2020; Expiration date: October 16, 2021

### Learning Objectives

Upon completion of this activity, participants will be able to:

- Describe the impact of IEC- and *tarP*-harboring phages on household transmission of LA-MRSA in the North Denmark Region during 2004–2011, according to whole-genome sequencing and epidemiologic investigations
- Determine the association of IEC- and *tarP*-harboring phages in LA-MRSA in the North Denmark Region during 2004–2011 with spread in the general population, according to an analysis of all Danish patients who had an episode of LA-MRSA infection during 2007–2018
- Identify clinical and public health implications of the effect of IEC- and *tarP*-harboring phages on household transmission of LA-MRSA in the North Denmark Region during 2004–2011, and of their association with spread into the general population

### CME Editor

**Dana C. Dolan, BS,** Copyeditor, Emerging Infectious Diseases. *Disclosure: Dana C. Dolan, BS, has disclosed no relevant financial relationships.*

### CME Author

**Laurie Barclay, MD,** freelance writer and reviewer, Medscape, LLC. *Disclosure: Laurie Barclay, MD, has disclosed no relevant financial relationships.*

### Authors

*Disclosures:* ***Raphael N. Sieber, PhD; Tinna Urth, MPH; Andreas Petersen, PhD; Camilla Møller, MD, PhD; Lance B. Price, PhD; Robert L. Skov, MD; Anders Larsen, PhD; Marc Stegger, PhD;*** *and* ***Jesper Larsen, PhD,*** *have disclosed no relevant financial relationships.*

Livestock-associated methicillin-resistant *Staphylococcus aureus* (LA-MRSA) clonal complex (CC) 398 is a major cause of zoonotic disease in Denmark and several other countries in Europe that have industrial pig production (*1*–*3*). In Denmark, the prevalence of LA-MRSA CC398 in pig farms increased from 3.5% in 2008 to »90% in 2018, when LA-MRSA CC398 accounted for 21% of all human MRSA infections (*3*). Most LA-MRSA CC398 infections occur in young and otherwise healthy livestock workers and their household contacts (*3*–*5*). Although LA-MRSA CC398 seems to be poorly adapted for human-to-human transmission (*6*), it is nonetheless able to spread to and cause serious illness and even death in elderly and immunocompromised persons in community and healthcare settings (*3*–*5*).

*S*. *aureus* uses a diverse range of immune-evasive strategies to maintain a lifelong relationship with the human host, many of which are encoded on phages and other mobile genetic elements (*7*). Of note, human *S*. *aureus* clones harbor a genetic element, the immune evasion cluster (IEC), on a 44-kb ΦSa3int prophage that is stably integrated into the *hlb* gene on the bacterial chromosome (*8*,*9*). The IEC element encodes >1 immune modulators, including staphylococcal complement inhibitor, chemotaxis inhibitory protein of staphylococci, staphylokinase, staphylococcal enterotoxin A, and staphylococcal enterotoxin P, which interact specifically with components of the human innate immune system (*7*). LA-MRSA CC398 isolates are descendants of a human variant of *S*. *aureus* CC398 but have lost the ΦSa3int prophage and the associated IEC element in connection with the host switch event (*10*); this change provides a potential explanation for the observed relatively low human-to-human transmissibility of LA-MRSA CC398 (*6*). However, some studies have shown that LA-MRSA CC398 might be capable of readapting to the human host through acquisition of phage-encoded immune modulators. For example, a Denmark study showed that 6% of human LA-MRSA CC398 isolates collected during 2004–2011 harbored the IEC element, whereas a more recent study revealed that 40% of LA-MRSA CC398 isolates from pigs in Denmark produce another phage-encoded immune modulator enzyme known as TarP, which enables *S*. *aureus* to subvert antibody-mediated immune recognition by altering a dominant cell surface epitope known as wall teichoic acids (WTA) (*3*,*11*).

These earlier findings raise important questions about the source and dynamics of phages encoding IEC and TarP in LA-MRSA CC398 and their role in host adaptation. In this study, we sequenced and compared a collection of epidemiologically well-characterized LA-MRSA CC398 isolates from humans and pigs in North Denmark Region to determine their population structure and the contribution of IEC and TarP to household transmission. We also used national surveillance data to further investigate whether IEC plays a role during spread of LA-MRSA CC398 into the community and healthcare settings.

Data used in this study were collected as part of the national MRSA surveillance program, as approved by the Danish Data Protection Agency (protocol no. 2001-14-0021). The National Committee on Health Research Ethics waived the need for approval and informed consent because data and biologic material were fully anonymized and collected in compliance with national legislation on statutory notification of MRSA in humans.

## Methods

### Study Population

North Denmark Region (≈7,900 km^2^) is a semirural area in northwest Denmark that had a population of ≈580,000 persons and ≈3 million pigs in 2011 (http://www.statbank.dk). We identified the study population using surveillance data collected by the regional infection control staff in North Denmark Region; it included all patients colonized or infected with LA-MRSA CC398 during 2004–2011, as well as all their household contacts who tested negative during the same period. We interviewed all of these persons to obtain relevant information, including sex, age, livestock contact, residential address, and workplace, and assigned each to 1 of 3 categories: livestock-exposed persons (direct contact); household contacts of livestock-exposed persons (indirect contact); and persons not connected to livestock production (no contact). We defined livestock-exposed persons as primary cases in their households, and the index person as the primary case in households with no connection to livestock production.

### Study Isolates and Data Aggregation

The study isolates comprised 96 human LA-MRSA CC398 isolates from North Denmark Region collected by regional infection control staff during 2004–2011 and 45 LA-MRSA CC398 isolates collected from pigs (1 isolate per farm) in North Denmark Region in 2014 (Appendix 1 Table 1). The human isolates have been characterized previously for *spa* type, presence of IEC, and antimicrobial susceptibilities (*3*); the pig isolates originated from a study investigating the population structure and dynamics of LA-MRSA CC398 in the pig population in Denmark (*12*). We used whole-genome sequencing and bioinformatics analyses to study the phylogenetic distribution, genetic diversity, and host association of IEC-harboring and *tarP*-harboring prophages among the 141 study isolates (Appendix 2).

### Analysis of National Surveillance Data

MRSA has been notifiable in Denmark since November 2006. As part of the national MRSA surveillance program, local clinical microbiology departments perform *S*. *aureus* identification and antimicrobial susceptibility testing and submit all confirmed MRSA isolates to the National Reference Laboratory for Antimicrobial Resistance at Statens Serum Institut (Copenhagen, Denmark), which collects patient information from general practitioners and assesses the *spa* type or the clonal complex and the presence or absence of the IEC element. The following data are collected for each case: sex, age, livestock contact, residential address, indication for testing (screening or infection), and hospitalization dates. Cases without direct or indirect livestock contact are defined as healthcare-onset (HO) if the culture is obtained >48 hours after admission; healthcare-associated community-onset (HACO) if the person has had contact with the healthcare setting within the preceding 12 months or the culture is obtained within the first 48 hours after admission; or community-onset (CO) if no other criteria are met. For this study, we retrieved the following information about all patients in Denmark who had an episode of LA-MRSA CC398 infection during January 2007–December 2018 (n = 1,545): sex; age; direct, indirect, or no livestock contact; location of disease onset (e.g., HO, HACO, or CO); and presence or absence of the IEC element in the corresponding LA-MRSA CC398 isolate. We calculated the excess number of clinical cases due to increased spread of IEC-positive isolates into a given patient group of interest as the total number of cases in the patient group of interest multiplied by the difference between the proportion of IEC-positive isolates in the patient group of interest (the sink) and the patient group with direct contact to livestock (the source).

### Statistical Analysis

We used Fisher exact test to analyze categorical data and Student t test to analyze continuous data (GraphPad Prism version 5; GraphPad, https://www.graphpad.com). We reported prevalence differences between different groups as prevalence ratios (PRs) and 95% CIs. The significance level was set at α = 0.05.

## Results

### Study Population

A total of 96 patients were colonized or infected with LA-MRSA CC398 in North Denmark Region during 2004–2011, including 67 primary cases and 29 secondary cases from 65 households. A total of 71 household contacts tested negative. The 67 primary cases comprised 57 persons with direct animal contact, 2 with indirect animal contact, and 8 with no animal contact. Those with direct animal contact included 44 pig farm employees from 42 households and 23 animal farms (2 households each contained 2 pig farm employees), 3 mink farm employees from 3 households and animal farms, 1 cattle farm employee, 1 turkey farm employee, 1 pig veterinarian, 3 lorry drivers transporting pigs from 3 households, 2 pig abattoir workers from 2 households, and 2 craftsmen working in pig stables from 2 households. Persons with indirect contact were from 2 households (a wife and a child of pig farm employees who were never tested), and those with no contact were from 8 households.

### Distribution of IEC and *tarP* in LA-MRSA CC398 

Most of the human isolates collected from persons living in the same household clustered together, with an average pairwise single-nucleotide polymorphism (SNP) distance of 5.9 (range 0–18 SNPs) and were genotypically homogeneous with respect to presence of specific IEC-harboring and *tarP*-harboring prophages (Appendix 2 Figure). Furthermore, human isolates from different households connected to the same animal farm also tended to cluster together but were genotypically more diverse than isolates from the same household (19.6 SNPs [range 0–156 SNPs] in different households versus 6.7 SNPs [range 0–18 SNPs] in the same household; p = 0.027). Pig isolates collected from unique farms were widely distributed across the phylogeny; the average pairwise SNP distance was 95.4 (range 3–224 SNPs).

**Figure 1:**
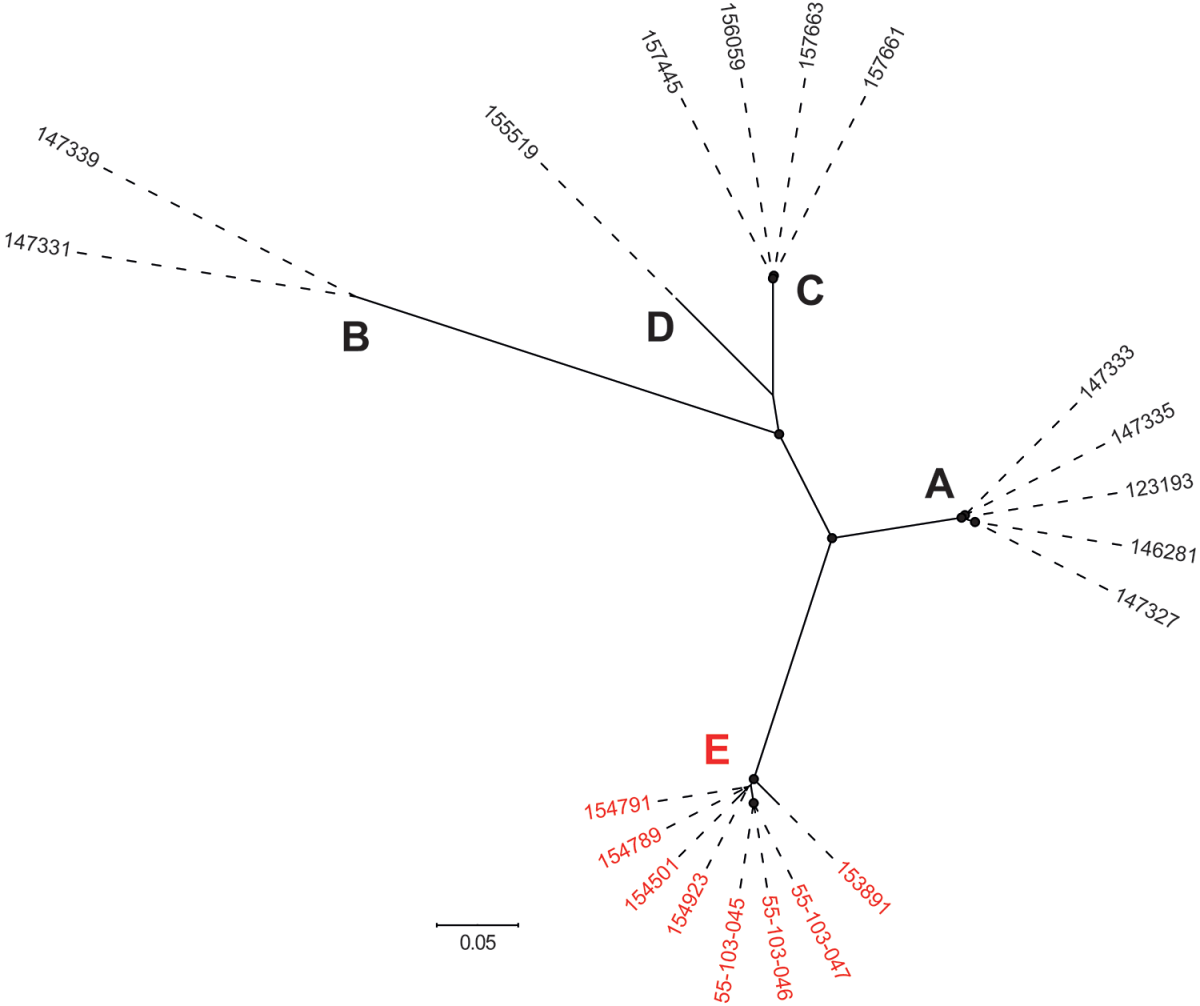
Maximum-likelihood phylogeny showing the genetic diversity of the 20 IEC-harboring ΦSa3int prophages identified in livestock-associated methicillin-resistant *Staphylococcus aureus* CC398 isolates from North Denmark Region, Denmark. Capital letters indicate phylogenetic clusters (A–E). Red text indicates ΦSa3int prophages harboring both immune evasion cluster and *tarP* (cluster E). The phylogeny was estimated for 795 high-quality core SNPs. Filled circles at nodes indicate bootstrap values >90%. Scale bar represents number of nucleotide substitutions per variable site.

We identified IEC in 20 isolates, which clustered within a closely related clade (Appendix 2 Figure). The IEC-harboring ΦSa3int prophages could be divided into 6 variants (I–VI) and 5 phylogenetic clusters (A–E) on the basis of their phylogenetic relationship, IEC type, and chromosomal integration site (Table 1; Figure 1). Each of the 6 ΦSa3int variants was unique to isolates from a single household. The 6 households comprised 4 pig farm employees (ΦSa3int-I–IV), a pig veterinarian (ΦSa3int-V), and a mink farm employee (ΦSa3int-VI), and their household contacts (Table 1). We also detected ΦSa3int-VI in 3 pig isolates that were closely related to the isolates from the mink farm employee’s household; the prophage was furthermore highly similar to ΦSa3int-V present in the isolate from the pig veterinarian but integrated into a different part of the chromosome (Table 1).

**Table 1:** Distribution of IEC-harboring ΦSa3int prophages among livestock-associated methicillin-resistant *Staphylococcus aureus* CC398 isolates from North Denmark Region, Denmark*

Variant	Phylogenetic cluster†	IEC type	Integration site‡	*tarP*	Isolate origin	Household ID	Farm ID
I	A	B	0723–0724	–	1 pig farm employee and 4 household members	H02	F01
II	B	E	2238	–	1 pig farm employee and 1 household member	H30	F01
III	C	B	2059 (*hlb*)	–	1 pig farm employee and 3 household members	H63	F04
IV	D	B	2591 (*cidA*)	–	1 pig farm employee	H51	F07
V	E	F	2059 (*hlb*)	+	1 pig veterinarian	H46	None
VI	E	F	2644	+	1 mink farm employee and 3 household members	H49	F21
	E	F	2644	+	Pig	NA	A
	E	F	2644	+	Pig	NA	B
	E	F	2644	+	Pig	NA	C

*Allocation of ΦSa3int prophages into variants (ΦSa3int-I to ΦSa3int-VI) was based on their phylogenetic relationship, IEC type, and chromosomal integration site. CC, clonal complex; ID, identification; IEC, immune evasion cluster; NA, not applicable; –, negative, +, positive.  †Phylogenetic clusters are illustrated in Figure 1. ‡Numbers refer to annotated genes in reference strain S0385 (GenBank accession no. NC_017333).

We identified the *tarP* gene in 45 isolates, which were more widely distributed across the phylogeny than the IEC-positive isolates (Appendix 2 Figure). Analysis of the genetically linked *int* genes showed that *tarP* was carried on 5 different prophages (ΦSa1int, ΦSa3int, ΦSa7int, ΦSa9int, and a prophage with an untypeable *int* gene hereafter referred to as ΦUT1). BLASTN searches (https://blast.ncbi.nlm.nih.gov/Blast.cgi) showed that the *int* gene in ΦUT1 was 100% identical to the *int* gene in the bovine *S*. *aureus* phage ΦDW2 (GenBank accession no. KJ140076). We found Φ*tarP*-Sa9int exclusively in pig isolates (n = 15), whereas the other *tarP*-harboring prophages were identified in 25 human isolates from 16 households and in 5 pig isolates.

IEC was more prevalent in human isolates (17/96; 18%) than in pig isolates (3/45; 6.7%), although the difference was not statistically significant (PR 2.66, 95% CI 0.90–8.26; p = 0.12). By contrast, *tarP* was significantly less prevalent in human isolates (25/96; 26%) than in pig isolates (20/45; 44%) (PR 0.59, 95% CI 0.37–0.95; p = 0.034). The IEC element in the 3 pig isolates was genetically linked to *tarP* on ΦSa3int-V and ΦSa3int-VI (Table 1; Appendix 2 Figure).

### Role of IEC and *tarP* during Household Transmission of LA-MRSA CC398 in North Denmark Region

Household transmission of LA-MRSA CC398 was based on detection of secondary cases of colonization or infection with isolates that were closely related and genotypically indistinguishable from the isolate of the primary case. Households consisting of 1 person (n = 26) and households with >2 persons reporting direct animal contact (n = 2) were excluded from both analyses. In addition, 1 household with a mixed population of IEC-positive and IEC-negative isolates was excluded from the corresponding analysis.

IEC-positive isolates were present in 14% (5/36) of the eligible households. Secondary transmission occurred more often in IEC-positive households (4/5; 80%) than in IEC-negative households (10/31; 32%), although the difference was not statistically significant (PR 2.48, 95% CI 1.04–4.64; p = 0.064). The proportion of secondary cases was significantly higher in IEC-positive households (65%; 11/17) than in IEC-negative households (22%; 16/74) (PR 2.99, 95% CI 1.65–5.12; p = 0.0010). Isolates carrying *tarP* were present in 32% (12/37) of the eligible households. We saw no differences in occurrence of secondary transmission between *tarP*-positive households (5/12; 42%) and *tarP*-negative households (9/25; 36%) (PR 1.16, 95% CI 0.47–2.55; p = 1.00) or in the proportion of secondary cases between *tarP*-positive households (10/33; 30%) and *tarP*-negative households (17/62; 27%) (PR 1.28, 95% CI 0.65– 2.42; p = 0.48). The average number of household contacts per household, excluding the primary case, did not differ significantly between IEC-positive (3.4; range 2–5) and IEC-negative households (2.4; range 1–7; p = 0.17) or between *tarP*-positive (2.8; range 1–7) and *tarP*-negative households (2.5; range, 1–5; p = 0.62). These findings indicate that IEC, but not *tarP*, facilitates household transmission of LA-MRSA CC398.

### Prevalence of IEC among LA-MRSA CC398 Isolates in Persons with No Livestock Contact

We investigated whether IEC also plays a role during spread of LA-MRSA CC398 in the general population, on the assumption that persons with direct livestock contact serve as the source of transmission to their household contacts (i.e., persons with indirect livestock contact) and into the local community, through which the bacterium is transmitted into healthcare settings. The analysis included 1,545 isolates from patients in 4 groups: patients with direct livestock contact (n = 727); patients with indirect livestock contact (n = 256); patients with CO infection (n = 383); and patients with HO/HACO infection (n = 179). The results showed that the proportion of IEC-positive isolates increased along this hypothetical transmission chain, from 3.4% in patients with direct contact to livestock to 6.3% (PR 1.82, 95% CI 0.99–3.31; p = 0.068) in patients with indirect contact to livestock, 7.1% (PR 2.05, 95% CI 1.21–3.46; p = 0.010) in patients with CO infection, and 11% (PR 3.25, 95% CI 1.85–5.65; p = 0.0001) in patients with HO/HACO infection (Table 2). The excess number of clinical cases attributable to increased spread of IEC-positive isolates ranged from 7 (2.8%) among persons with indirect livestock contact to 14 in both the community (3.6%) and healthcare (7.7%) settings (Table 2). These findings demonstrate an association of IEC with increased human-to-human transmission and excess disease burden of LA-MRSA CC398.

**Table 2:** Patient characteristics and presence of IEC among livestock-associated methicillin-resistant *Staphylococcus aureus* CC398 cases and isolates, North Denmark Region, Denmark*

Patient group	No. isolates	Male:female ratio	Median age, y (range)	% IEC	No. IEC-related excess cases (% all cases)	Prevalence ratio (95% CI)	p value
Patients with livestock contact							
Direct contact	727	3.2	33 (0–93)	3.4	Referent	Referent	Referent
Indirect contact	256	0.59	22 (0–91)	6.3	7 (2.8)	1.82 (0.99–3.31)	0.068
Patients with CO infection	383	1.0	52 (0–98)	7.1	14 (3.6)	2.05 (1.21–3.46)	0.010
Patients with HO/HACO infection	179	1.1	66 (0–97)	11	14 (7.7)	3.25 (1.85–5.65)	0.0001

*CC, clonal complex; CO, community-onset; HACO, healthcare-associated community-onset; HO, healthcare-onset; IEC, immune evasion cluster.

**Figure 2:**
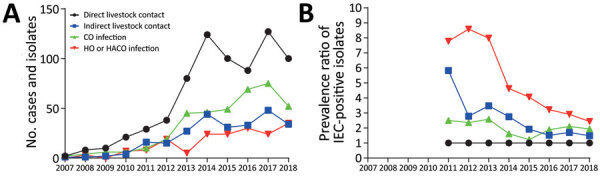
Temporal trends of livestock-associated methicillin-resistant *Staphylococcus aureus* infections in Denmark. A) Annual number of cases and isolates. B) Proportion of IEC-positive isolates in patients with indirect livestock contact, CO, and HO/HACO infection compared with patients who had direct contact with livestock. Data are shown as prevalence ratios (5-year rolling averages). CO, community-onset; HACO, healthcare-associated community-onset; HO, healthcare-onset; IEC, immune evasion cluster.

If IEC-positive isolates become fixed (i.e., self-sustainable) in the general population, we expect their proportion to increase over time in patients with CO and HO/HACO infections, compared with patients with direct contact to livestock. However, although the number of LA-MRSA CC398 infections increased in all 4 patient groups during 2007–2018 (Figure 2, panel A), the prevalence ratio of IEC-positive isolates among patients with CO and HO/HACO infections either decreased or remained relatively stable over the years (Figure 2, panel B).

## Discussion

Successful spread of *S*. *aureus* in humans depends upon the bacterium’s ability to survive and multiply in newly colonized persons. For example, *S*. *aureus* must compete with other bacteria and avoid the innate and adaptive immune defenses of the skin and nasal environments. In this study, we have shown that acquisition of IEC, but not *tarP*, is associated with increased household transmission of LA-MRSA CC398 and excess spread into the community and healthcare settings. The findings should be interpreted with some caution because of the small number of households and cases analyzed, and more follow-up studies should be done to further evaluate the relative contribution of IEC and other risk factors, such as household size (*13*), to the spread of LA-MRSA CC398 in humans.

Animal and human studies have shown that IEC is ubiquitous in human *S*. *aureus* clones, whereas it is consistently absent in livestock-associated *S*. *aureus* clones (*9*,*10*). Staphylococcal complement inhibitor and other IEC-encoded immune modulators have human-specific activities toward central components of the innate immune response, such as neutrophils and complements, and are produced in both healthy carriers and patients with *S*. *aureus* infection (*14*–*16*). Our findings suggest that IEC promotes survival of *S*. *aureus* in the human host, which in turn is expected to increase the likelihood of human-to-human transmission.

Nonetheless, the disease burden of IEC-positive LA-MRSA CC398 isolates in the community and healthcare settings remains relatively low in Denmark. We saw no suggestion that they will become fixed in the general population; van Alen et al. reached the same conclusion after finding a low, albeit slightly increasing, prevalence of IEC among LA-MRSA CC398 isolates from hospital patients during 2000–2015, ranging from 1.1% during 2000–2006 to 3.9% during 2007–2015 (*17*). Research indicates several possible reasons why IEC-positive LA-MRSA CC398 isolates have not become self-sustainable in humans. First, our findings showed that the 20 IEC-positive isolates from North Denmark Region clustered within a closely related clade, indicating that not all LA-MRSA CC398 isolates have the same ability to acquire ΦSa3int phages. Second, a recent study (*18*) has shown that LA-MRSA CC398 carries substitutions at the usual attachment site (*att*B) located within the *hlb* gene, which interferes with phage integration and might explain why ΦSa3int phages often integrate into other genomic regions than the *hlb* gene in LA-MRSA CC398 through recombination between the phage attachment site (*att*P) and alternative *att*B sites, as demonstrated in our study and others (*17*–*19*). Third, it is possible that LA-MRSA CC398 has undergone other genetic changes, in addition to losing the IEC-harboring ΦSa3int prophage during the human-to-animal host switch event, that are beneficial in the livestock reservoir but detrimental in the human host. For example, LA-MRSA CC398 isolates from Denmark have acquired several mobile genetic elements encoding resistance to a wide range of the most frequently used antimicrobial drugs in pigs, including β-lactams, aminoglycosides, macrolides, tetracyclines, and zinc (*12*), which are likely to exert a fitness cost outside the livestock reservoir (*20*). This possibility is supported by our recent finding that LA-MRSA CC398 isolates from hospital patients carry far fewer antimicrobial resistance genes of veterinary importance than LA-MRSA CC398 isolates from pigs (*21*).

The widespread distribution of IEC among human *S*. *aureus* clones, the phylogenetic clustering of IEC-harboring ΦSa3int prophages in LA-MRSA CC398 isolates from the same household, and the low prevalence of IEC in LA-MRSA CC398 isolates from pigs suggest that acquisition of IEC by LA-MRSA CC398 mainly occurs through transfer of ΦSa3int phages from human *S*. *aureus* donors circulating in households. In support of this view, we found little evidence for phage transfer or transmission of IEC-positive isolates between pigs and humans. An exception was a possible transmission chain involving 3 IEC-positive isolates from pigs and 4 isolates from a mink farm employee and his household members and a possible phage transfer event between this cluster of isolates and an isolate from a pig veterinarian. Of note, the IEC-harboring ΦSa3int prophage found in the 3 pig isolates also encoded TarP, thus raising the possibility that IEC can be passively maintained in the pig population by co-selection for other traits.

TarP-mediated protection against anti-WTA antibodies did not seem to influence household transmission of LA-MRSA CC398 in our study despite the fact that anti-staphylococcal antibodies are present at high levels in serum and nasal secretions of both persistent *S*. *aureus* carriers and noncarriers (*15*,*16*). Instead, there is evidence that the much more widely distributed staphylococcal protein A (SpA), which is produced by all known human and livestock-associated *S*. *aureus* clones, is sufficient for escaping the adaptive immune response. SpA contains several immunoglobulin-binding domains capable of binding both the Fcγ of IgG antibodies and the Fab of V_H_3-idiotype antibodies, thereby limiting opsonophagocytosis and broad-spectrum antibody responses to other secreted and surface-bound antigens during *S*. *aureus* colonization and infection (*22*–*30*).

In summary, our study suggests that acquisition of IEC, but not *tarP*, is associated with increased household transmission of LA-MRSA CC398 and spillover into the community and healthcare settings, which might also explain why IEC is widespread among human *S*. *aureus* clones. Despite these findings, the attributable disease burden remains relatively low in Denmark, and we found no evidence to suggest that they have become self-sustainable in the general population. However, the dynamic nature of *S*. *aureus* genome evolution and host adaptability, as documented here and elsewhere, underscores the need for continued surveillance at the human–animal interface to detect evolutionary as well as epidemiologic changes that affect public health.

Appendix 1 Isolates and genes from study of transmission of livestock-associated methicillin-resistant *Staphylococcus aureus* infections in Denmark.

Appendix 2 Additional information about transmission of livestock-associated methicillin-resistant *Staphylococcus aureus* infections in Denmark.

## Earning CME Credit

To obtain credit, you should first read the journal article. After reading the article, you should be able to answer the following, related, multiple-choice questions. To complete the questions (with a minimum 75% passing score) and earn continuing medical education (CME) credit, please go to http://www.medscape.org/journal/eid. Credit cannot be obtained for tests completed on paper, although you may use the worksheet below to keep a record of your answers. 

You must be a registered user on http://www.medscape.org. If you are not registered on http://www.medscape.org, please click on the “Register” link on the right hand side of the website. 

Only one answer is correct for each question. Once you successfully answer all post-test questions, you will be able to view and/or print your certificate. For questions regarding this activity, contact the accredited provider, CME@medscape.net. For technical assistance, contact CME@medscape.net. American Medical Association’s Physician’s Recognition Award (AMA PRA) credits are accepted in the US as evidence of participation in CME activities. For further information on this award, please go to https://www.ama-assn.org. The AMA has determined that physicians not licensed in the US who participate in this CME activity are eligible for *AMA PRA* *Category 1 Credits™*. Through agreements that the AMA has made with agencies in some countries, AMA PRA credit may be acceptable as evidence of participation in CME activities. If you are not licensed in the US, please complete the questions online, print the AMA PRA CME credit certificate, and present it to your national medical association for review.

## Article Title:  Phage-Mediated Immune Evasion and Transmission of Livestock-Associated Methicillin-Resistant *Staphylococcus aureus* in Humans

## CME Questions

1. **You are advising a public health department about the likelihood of livestock-associated methicillin-resistant *Staphylococcus aureus* (LA-MRSA) transmission. According to the whole-genome sequencing and epidemiologic study by Sieber and colleagues, which of the following statements about the impact of immune evasion cluster (IEC)- and *tarP*-harboring phages on household transmission of LA-MRSA in the North Denmark Region during 2004-2011 is correct?**
  - *tarP*, but not IEC, facilitates household transmission of LA-MRSA CC398
  - The proportion of secondary cases was significantly higher in IEC-negative than in IEC-positive households
  - Secondary transmission occurred more often in IEC-positive than in IEC-negative households, but the difference was not statistically significant (80% vs 32%; p = 0.064)
  - *tarP*-204–positive vs *tarP*-negative households had significantly greater secondary transmission and proportion of secondary cases
2. **According to the analysis by Sieber and colleagues of all Danish patients who had an episode of LA-MRSA infection during 2007–2018, which of the following statements about the association of IEC- and *tarP*-harboring phages in LA-MRSA in the North Denmark Region during 2004–2011 with spread in the general population is correct?**
  - Horizontal acquisition of IEC in the human host was associated with increased household transmission of LA-MRSA CC398 and spillover into the community and healthcare settings
  - The findings suggest that IEC-positive LA-MRSA isolates have become self-sustainable in the general population
  - Proportion of IEC-positive isolates was highest in cases with direct livestock contact and lowest in cases with healthcare-onset (HO) or healthcare-associated community-onset (HACO) infection
  - The prevalence ratio of IEC-positive isolates among patients with CO and HO/HACO infections increased over time
3. **According to the genetic and epidemiologic study by Sieber and colleagues, which of the following statements about clinical and public health implications of the effect of IEC- and *tarP*-harboring phages on household transmission of LA-MRSA in the North Denmark Region during 2004–2011, and of their association with spread into the general population, is correct?**
  - The findings suggest that all LA-MRSA CC398 isolates have the same ability to acquire ΦSa3int phages
  - Acquisition of IEC by LA-MRSA CC398 rarely occurs through transfer of ΦSa3int phages from human S aureus donors circulating in the respective households
  - TarP-mediated protection against anti-wall teichoic acid (WTA) antibodies increased household transmission of LA-MRSA CC398
  - Changing S aureus genome evolution and host adaptability mandate ongoing human-animal surveillance to detect evolutionary and epidemiologic changes that may affect public health
