# Supplementary material for: Phage-Mediated Immune Evasion and Transmission of Livestock-Associated Methicillin-Resistant Staphylococcus aureus in Humans
Source: Emerg Infect Dis. 2020 Nov;26(11):2578–85. doi: 10.3201/eid2611.201442 (PMC7588543; doi:10.3201/eid2611.201442)
Supplement: Appendix 2 — Additional information about transmission of livestock-associated methicillin-resistant Staphylococcus aureus infections in Denmark. [file 20-1442-Techapp-s2.pdf]

Article DOI: https://doi.org/10.3201/eid2611.201442

Phage-Mediated Immune Evasion and Transmission of Livestock-Associated Methicillin-Resistant *Staphylococcus aureus* in Humans

Appendix 2

Methods

Whole-Genome Sequencing

Whole-genome sequences were available for all 45 pig isolates and 21 of the human isolates (*1*–*3*) and were downloaded from the NCBI Sequence Read Archive via BioProject accession nos. PRJEB19505, PRJEB25608, and PRJNA274898 (Appendix Table 1). The remaining 75 human isolates were subjected to DNA extraction, library preparation, and whole-genome sequencing on different Illumina platforms as described previously (*2*,*3*). The whole-genome sequence data from this study have been submitted to the NCBI Sequence Read Archive under BioProject accession no. PRJNA613886 (Appendix Table 1).

Sequence Analysis

De novo assemblies were generated with SPAdes (*4*). The ΦSa3int prophage (ΦNM3) and the *scn, chp, sak,* and *sea* genes present in *S. aureus* strain Newman (GenBank accession no. NC_009641) and the *sep* and *tarP* genes present in *S. aureus* strain N315 (GenBank accession no. BA000018.3) were used as queries in BLASTN searches against the de novo assemblies. The genetic location and orientation of the ΦSa3int prophage in the study isolates were determined by performing BLASTN searches against the LA-MRSA CC398 reference strain S0385 (GenBank accession no. NC_017333) using the first 500 nucleotides flanking the left (attL) and right (attR) attachment sites, respectively, as queries (*5*). IEC elements carrying different combinations of the *scn, chp, sak, sea,* and *sep* genes were assigned into unique types using a previously published scheme (*6*). Phages encoding TarP were divided into previously defined integrase (*int*) classes according to the nucleotide sequence of the *int* gene (*7*), which is located in a highly conserved position approximately 300 bp upstream of *tarP* (*8*). Sequences were compared with a reference collection of *int* genes (Appendix Table

2) using BLASTN, and genes with 100% match to length and >95% identity match were classified as present. Sequences that did not produce a significant hit were used as queries in BLASTN searches against the NCBI nucleotide collection (accessed 2019 Nov 27).

Phylogenetic Analysis

Sequence reads from all study isolates were mapped against LA-MRSA CC398 strain S0385 (GenBank accession no. NC_017333), and single nucleotide polymorphism (SNP) calling were carried out using the NASP pipeline as described previously (*3*). Recombination was removed from the SNP alignment using Gubbins (*9*), and the remaining SNPs in the core genome were used to construct a maximum-likelihood tree using PhyML with a GTR model of nucleotide substitution and 100 bootstrap replicates (*10,11*).

The phylogenetic relationship between ΦSa3int prophages were investigated in a separate analysis, in which sequence reads from the subset of study isolates that harbored the ΦSa3int prophage were mapped against *S. aureus* strain Newman (GenBank accession no. NC_009641) followed by SNP calling and manual removal of SNPs located outside the ΦNM3 prophage (corresponding to nucleotide positions 2,088,220–2,132,279 in *S. aureus* strain Newman). The remaining SNPs were used to construct a maximum-likelihood tree as described above.

References

1. Sieber RN, Skov RL, Nielsen J, Schulz J, Price LB, Aarestrup FM, et al. Drivers and dynamics of methicillin-resistant livestock-associated *Staphylococcus aureus* CC398 in pigs and humans in Denmark. MBio. 2018;9:e02142-18. PubMed https://doi.org/10.1128/mBio.02142-18

2. Price LB, Stegger M, Hasman H, Aziz M, Larsen J, Andersen PS, et al. *Staphylococcus aureus* CC398: host adaptation and emergence of methicillin resistance in livestock. MBio. 2012;3:e00305-11. PubMed https://doi.org/10.1128/mBio.00305-11

3. Larsen J, Petersen A, Larsen AR, Sieber RN, Stegger M, Koch A, et al.; Danish MRSA Study Group. Emergence of livestock-associated methicillin-resistant *Staphylococcus aureus* bloodstream infections in Denmark. Clin Infect Dis. 2017;65:1072–6. PubMed https://doi.org/10.1093/cid/cix504

4. Bankevich A, Nurk S, Antipov D, Gurevich AA, Dvorkin M, Kulikov AS, et al. SPAdes: a new genome assembly algorithm and its applications to single-cell sequencing. J Comput Biol. 2012;19:455–77. PubMed https://doi.org/10.1089/cmb.2012.0021

5. Bae T, Baba T, Hiramatsu K, Schneewind O. Prophages of *Staphylococcus aureus* Newman and their contribution to virulence. Mol Microbiol. 2006;62:1035–47. PubMed https://doi.org/10.1111/j.1365-2958.2006.05441.x

6. van Wamel WJ, Rooijakkers SH, Ruyken M, van Kessel KP, van Strijp JA. The innate immune modulators staphylococcal complement inhibitor and chemotaxis inhibitory protein of *Staphylococcus aureus* are located on beta-hemolysin-converting bacteriophages. J Bacteriol. 2006;188:1310–5. PubMed https://doi.org/10.1128/JB.188.4.1310-1315.2006

7. Goerke C, Pantucek R, Holtfreter S, Schulte B, Zink M, Grumann D, et al. Diversity of prophages in dominant *Staphylococcus aureus* clonal lineages. J Bacteriol. 2009;191:3462–8. PubMed https://doi.org/10.1128/JB.01804-08

8. Gerlach D, Guo Y, De Castro C, Kim SH, Schlatterer K, Xu FF, et al. Methicillin-resistant *Staphylococcus aureus* alters cell wall glycosylation to evade immunity. Nature. 2018;563:705–9. PubMed https://doi.org/10.1038/s41586-018-0730-x

9. Croucher NJ, Page AJ, Connor TR, Delaney AJ, Keane JA, Bentley SD, et al. Rapid phylogenetic analysis of large samples of recombinant bacterial whole genome sequences using Gubbins. Nucleic Acids Res. 2015;43:e15. PubMed https://doi.org/10.1093/nar/gku1196

10. Guindon S, Gascuel O. A simple, fast, and accurate algorithm to estimate large phylogenies by maximum likelihood. Syst Biol. 2003;52:696–704. PubMed https://doi.org/10.1080/10635150390235520

11. Guindon S, Dufayard JF, Lefort V, Anisimova M, Hordijk W, Gascuel O. New algorithms and methods to estimate maximum-likelihood phylogenies: assessing the performance of PhyML 3.0. Syst Biol. 2010;59:307–21. PubMed https://doi.org/10.1093/sysbio/syq010

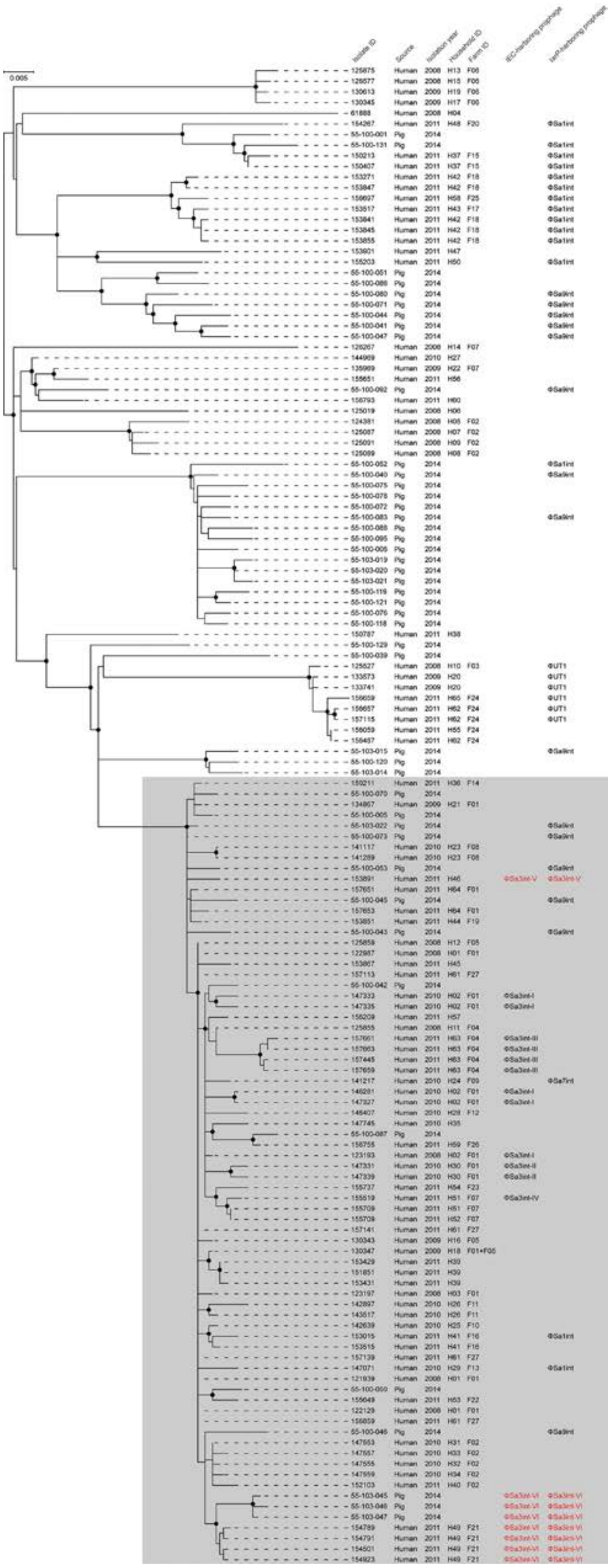

Appendix 2 Figure. Phylogenetic relationship among 141 livestock-associated methicillin-resistant *Staphylococcus aureus* CC398 (LA-MRSA CC398) isolates from North Denmark Region. The maximum-likelihood phylogeny was estimated for 1,636 high-quality core SNPs. The origin and presence of IEC and *tarP*-harboring phages are shown for each isolate. Red text indicates ΦSa3int

prophages harboring both IEC and *tarP*. The shaded area represents the clade comprising the 20 IEC-positive isolates. Filled circles at the nodes indicate bootstrap values >90%. Scale bar represents number of nucleotide substitutions per variable site.
